# Loss of expression of Syndecan-1 is associated with Tumor Recurrence, Metastatic Potential, and Poor Survival in patients with Colorectal carcinoma

**DOI:** 10.12669/pjms.37.1.2592

**Published:** 2021

**Authors:** Jaudah Al-Maghrabi

**Affiliations:** 1 Jaudah Al-Maghrabi, MD, FRCPC, FCAP. Department of Pathology, Faculty of Medicine, King Abdulaziz University, Jeddah, Saudi Arabia

**Keywords:** Syndecan-1, Metastasis, Survival, Colorectal carcinoma, CD138

## Abstract

**Objective:**

The loss of expression of syndecansyndecan-1 is associated with poor prognosis in many types of human cancer. The objective of this study was to evaluate the relation between syndecan-1 immunoexpression and several clinicopathological parameters in a subset of colorectal carcinoma (CRC) patients.

**Methods:**

Pathology tissue blocks of 202 primary tumors, 41 adenomas, and 37 normal colonic mucosae were used in this study. The cases diagnosed in the period 1995–2015 was included in the study. Immunohistochemistry analysis was performed using anti-CD138/syndecan-1 (B-A38) mouse monoclonal antibody. A semiquantitative method was used to score the syndecan-1 expression based on an evaluation of the percentage and intensity of the membranous and cytoplasmic expression. The data collected from Pathology Department at King Abdulaziz University Hospital, Jeddah, Saudi Arabia. This is a retrospective cohort study that was conducted from July 2018 until August 2019.

**Results:**

Loss of syndecan-1 immunoexpression was observed in 72 (42.6%), 5 (12.2%), and 3 (8.1%) cases of CRC, adenomas, and normal mucosae, respectively. Low expression of syndecan-1 showed an association with nodal (p=0.003) and distant (p=0.001) metastasis, lymphovascular invasion (p=0.001), and tumor recurrence (p=0.006). Low syndecan-1 expression were associated with short overall survival (OS) (log rank 4.019, p=0.045) and disease-free survival (DFS) probabilities (log rank 4.748, p=0.029).

**Conclusion:**

Loss of syndecan-1 immunoexpression is associated with metastatic potential, tumor recurrence and shorter survival in CRC and is considered a potential biomarker of poor prognosis in CRC patients.

## INTRODUCTION

Colorectal carcinoma (CRC) is a common gastrointestinal cancer in Saudi Arabia. It ranks as the first type among Saudi men and third among Saudi women.^1^ The molecular changes involved in CRC carcinogenesis are not yet clear. The mortality rate of CRC is high and caused primarily by invasion and metastasis of the tumor. Therefore, investigation of the molecular changes or triggering factors associated with invasion and metastasis in CRC is important and may contribute to changes in therapeutic approaches in the future.

Syndecan-1 (CD138) is a transmembrane proteoglycan and an important cell adhesion molecule.^2^ It is a member of the syndecan family and is predominantly expressed in epithelial cells. Syndecan-1 plays an important role in cell proliferation, adhesion, migration, and angiogenesis.^3^ A loss of immunoexpression of syndecan-1 has been found to be an indicator of poor prognosis in many human cancers, such as gastric, hepatocellular, lung, oral, ovarian and prostate.^4^-^10^

The prognostic significance of syndecan-1 remained controversial CRC with conflicting results.^11^-^14^ Thus, further evaluation is needed. The objective of this study was to evaluate the relation between the immunoexpression of syndecan-1 and several clinicopathological parameters in a subset of CRC patients from Saudi Arabia.

## METHODS

The patients’ data and histopathological material were collected from the Pathology Department at King Abdulaziz University Hospital, Jeddah, Saudi Arabia for the cases diagnosed in the period 1995–2015. Tumor stages were reviewed and reclassified according the cancer staging atlas of the American Joint Committee On Cancer.^15^

The pathology tissue blocks of 202 primary tumors, 41 adenomas, and 37 normal colonic mucosae were used in this study. The clinicopathological findings were collected including age, gender, tumor location, tumor size, tumour stage, or margin status, metastasis and lymphovascular invasion and shown in Table-I. The study was approved by the Research Committee of the Biomedical Ethics Unit at our institution. The procedures followed were in accordance with the Declaration of Helsinki of 1975, as revised in 2000. Informed written consent was obtained from each patient to obtain permission to utilize their pathological tissue specimens for laboratory studies. This is a retrospective cohort study that was conducted from July 2018 until August 2019.

**Table-I:** Clinicopathological parameters of cases (n=202).

Parameter		Number (%)
Age	<60 years	108 (53.5%)
	≥60 years	94 (46.5%)
Sex	Male	110 (55%)
	Female	92 (45%)
Tumor location	Right colon	52 (25.7%)
	Left colon	127 (62.9%)
	Rectum	23 (11.4%)
Tumor size	< 5cm	91 (45%)
	≥ 5cm	111 (55%)
Grade	Well-differentiated	43 (21.3%)
	Moderately-differentiated	133 (65.8%)
	Poorly-differentiated	26 (12.9%)
Primary tumor	T1	3 (1.5%)
	T2	32 (15.8%)
	T3	149 (73.8%)
	T4	18 (8.9%)
Nodal metastasis	Negative	111 (54.9 %)
	Positive	84 (41.6%)
	Cannot be assessed	7(3.5%)
Distant metastasis	Positive	57 (28.2%)
	Negative	145 (71.8%)
Lymphovascular invasion	Positive	31 (15.3%)
	Negative	171 (84.7%)
Margin status	Involved	10 (5%)
	Free	192 (95%)
Recurrence	Recurrence	63 (31.2%)
	No recurrence	139 (68.8%)

### Tissue Microarray

The tissue microarray was constructed as previously described.^16^,^17^ Pathology slides (haematoxylin and eosin-stained) of primary CRC, adenomas and normal colonic mucosa tissue were evaluated and selected areas were marked. Material from patients diagnosed in the period 1995-2015 was included in the study. The cases with areas that showed extensive necrosis, poor cellular preservation, crush artefacts, dominant stromal tissue, or autolytic changes were excluded from the study. Donor paraffin blocks that matched the chosen sections were utilized to get two cores of the selected tissue and then transferred to recipient blocks via a tissue microarray machine (TMA Master 1.14 SP3 from 3D Histech Ltd., Budapest, Hungary). Unstained 4-µm-thick sections were cut from the TMA blocks and utilized for immunohistochemistry studies.

### Immunohistochemistry

Immunocytochemistry was performed by utilizing CD138/syndecan-1 (B-A38) Mouse Monoclonal Antibody (Cell Marque™- a Sigma Aldrich® Company 6600 Sierra College Blvd. Rocklin, California 95677 United States). The antibody is optimally diluted to be compatible with VENTANA detection kits. An automated immunostainer (Ventana Bench Mark XT, Ventana Inc., Tucson, AZ) was used to perform the immunohistochemistry procedure. The positive control was a plasmacytoma tissue that is known to be CD138-positive. Negative controls were processed without adding the primary antibody.

### Evaluation of Syndecan-1 Immunostaining

The intensity of Syndecan-1 membranous/cytoplasmic staining was scored as follows; staining was scored from 0 to 3, where: 0 = negative; 1 = weak; 2 = moderate; and 3 = strong. The percentage of positively-stained cells was calculated as follows: (0, no stain; 1, 1–25%; 2, 26–50%; 3,> 50%). The intensity score was added to the percentage score to get a final score of 1-6. The total score was divided into two groups: a low-expression group (scores 0-2) and a high expression group (scores 3-6).

### Statistical analysis

The chi-squared test was used to test the differences between two groups of variables. The overall survival (OS) and disease-free survival (DFS) values were measured by the Kaplan-Meier method with the log-rank (Mantel-Cox) comparison test. DFS was calculated as the time from diagnosis to the appearance of recurrent disease (or date of the last seen disease-free appearance). Statistical analyses were performed using the SPSS® (IMB NY, USA) software package, version 20. P <0.05 was considered statistically significant.

### Ethical Approval

The Unit of the Biomedical Ethics, Research Committee, Faculty of Medicine, King Abdulaziz University, Jeddah, Saudi, Arabia, approved this study (Reference No. 1127-13).

## RESULTS

**Fig.1:**
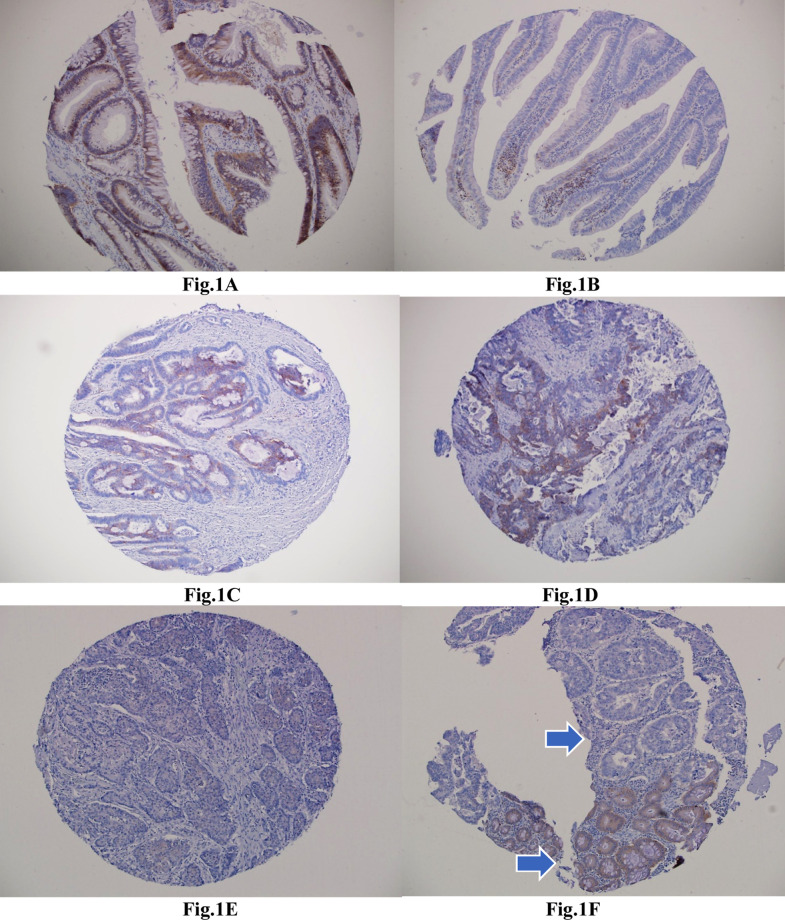
Syndecan-1 immunostaining (A) Sections from colonic adenoma show positive immunostaining, (B) Sections from colorectal adenoma show negative immunostaining with scattered positive plasma cells in the lamina propria as an internal positive control, (C) Section from a well differentiated colonic adenocarcinoma shows strong positive immunostaining, (D) Section from a moderately differentiated colonic adenocarcinoma shows moderate positive immunostaining, (E) Section from a poorly differentiated colonic adenocarcinoma shows negative immunostaining, (F) Section show a focus of colonic carcinoma with negative staining area (upper arrow), while a normal mucosa in the same sample show positive moderate staining (lower arrow)

The clinicopathological features of the cases are summarized in Table-I. high membranous/cytoplasmic staining of syndecan-1 was observed in 130 (57.4%), 36 (87.8%), and 34 (91.9%) cases of CRC, adenomas, and normal mucosae, respectively (Fig.1). Low expression staining was observed in 72 (42.6%), 5 (12.2%), and 3 (8.1%) cases of CRC, adenomas, and normal mucosae, respectively.

There is a statistically significant difference in syndecan-1 expression between CRC and adenomas (p=0.0058) and normal mucosae (p=0.0017). There was an association between low syndecan-1 expression and nodal (p=0.003) and distant (p=0.001) metastasis, lymphovascular invasion (p=0.001), and tumor recurrence (p=0.006). Low syndecan-1 expression was identified in 29% and 57% of well-differentiated and poorly differentiated tumors, respectively, but there was no statistically significant association with tumor grade (p=0.503).

**Fig.2:**
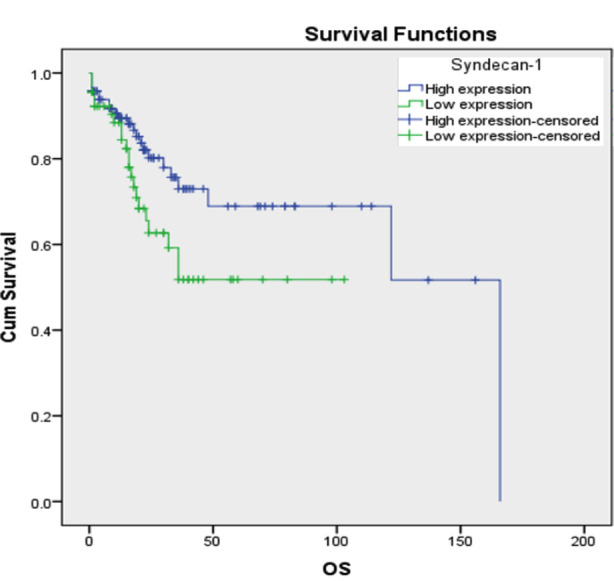
Overall survival curve (Kaplan Meier) in relation to Syndecan-1 immunoexpression in CRC patients. There is association between low Syndecan-1 expression and OS (Log rank 4.019, p=0.045).

**Fig.3:**
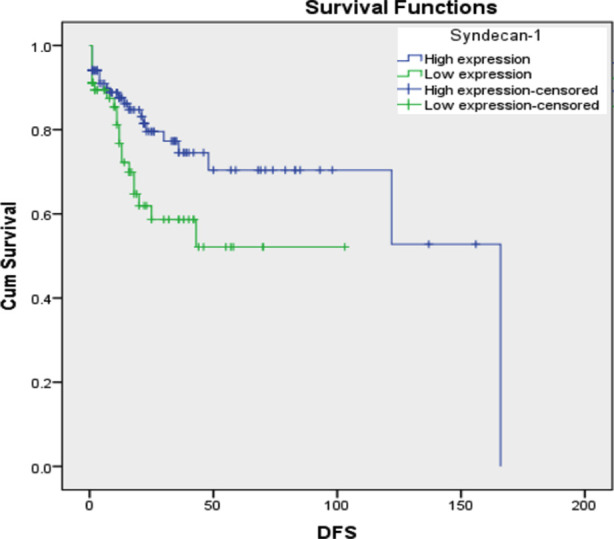
Disease-free survival curve (Kaplan Meier) in relation to Syndecan-1 immunoexpression in CRC patients There is association between low Syndecan-1 expression DFS (Log Rank 4.748, p=0.029).

Furthermore, syndecan-1 expression was not associated with age, gender, tumor location, tumor size, tumor stage, or margin status (Table-II). In the survival analysis, patients with low syndecan-1 expression tumors tended to have short OS (log rank 4.019, p=0.045) and DFS (log rank 4.748, p=0.029) (Fig.2 and 3).

**Table-II:** Distribution of Syndecan-1 immunoexpression in relation to clinicopathological parameters (n=202).

Parameter		Syndecan-1	Immunostaining	Test	p value

		Low expression	High expression		
Age	<60 years	39 (36.1%)	69 (63.9%)	Chi Square	0.500
	≥60 years	33(35.1%)	61(64.9%)		
Sex	Male	42(38.2%)	68(61.8%)	Chi Square	0.250
	Female	30 (32.6%)	62 (67.4%)		
Tumor location	Right colon	23 (44.2%)	29 (55.8%)	Chi Square	0.162
	Left colon	39( 30.7%)	88 (69.3%)		
	Rectum	10 (43.5%)	13 (56.5%)		
Tumor size	< 5cm	35 (38.5%)	56 (61.5%)	Chi Square	0.271
	≥ 5cm	37 (33.3%)	74 (66.7%)		
Grade	Well-differentiated	12 (29.3%)	29 (70.7%)	Chi Square	0.503
	Moderately-differentiated	48 (36.1%)	85 (63.9%)		
	Poorly-differentiated	12 (42.9%)	16 (57.1%)		
Primary tumor	T1	1 (33.3%)	2(66.7%)	Chi Square	0.320
	T2	7(21.9%)	25 (78.1%)		
	T3	56 (37.6%)	93 (62.4%)		
	T4	8 (44.4%)	10 (55.6%)		
Nodal metastasis	Positive	41 (48.8%)	43 (51.2%)	Chi Square	0.003
	Negative	28 (25.2%)	83 (74.8%)		
Distant metastasis	Positive	31(74%)	27 (26%)	Chi Square	0.001
	Negative	41 (28.5%)	103(71.5%)		
Lymphovascular invasion	Positive	19 (61.3%)	12 (38.7%)	Chi Square	0.001
	Negative	53 (31%)	118 (69%)		
Margin status	Involved	3 (30%)	7 (70%)	Chi Square	0.495
	Free	69 (35.9%)	123 (64.1%)		
Recurrence	Recurrence	31 (49.2%)	32(50.8%)	Chi Square	0.006
	No recurrence	41 (29.5%)	98 (70.5%)		

## DISCUSSION

The frequency of CRC varies markedly between countries, which is most likely due to differences in environmental and dietary factors. In Saudi Arabia, CRC is the most common cancer of the gastrointestinal tract.^1^ The main therapeutic approach for CRC patients is surgical resection. However, in metastatic and locally advanced disease, the therapeutic options are limited. Therefore, clear understanding of the molecular biology and the key factors that are involved in the progression of the disease is essential for improving the treatment approach of CRC patients.

Syndecans are membrane proteins that control cell proliferation, differentiation, adhesion, and migration.^18^ Syndecan-1 is an important adhesion cell molecule in this family of transmembrane proteoglycans. It plays an essential role in the binding of epithelial cells to the extracellular matrix.^12^ Syndecan-1 is usually expressed in stratified squamous and glandular epithelium and is considered an important molecule that is involved in the regulation of cell morphology, growth, and regeneration.^12^

In the current study, tissue microarray was utilized to evaluate immunoexpression in CRC. Low syndecan-1 expression was observed more frequently in CRC than adenomas and normal mucosae. loss of syndecan-1 immunoexpression was associated with metastatic potential, high recurrence rate and shorter survival in CRC which has a clinical significant in patients with CRC. A loss of epithelial expression of syndecan-1 has been found to be an indicator of poor prognosis in many human cancers.^4^-^10^,^19^-^22^

Low syndecan-1 expression was associated with nodal involvement, distant metastasis, lymphovascular invasion, and tumor recurrence. These results are consistent with other studies.^3^,^11^-^13^ However, Kim et al.^23^ found an association between syndecan-1 immunoexpression and tumor size, but not with other parameters, including nodal involvement, distant metastasis, and lymphovascular invasion. Peretti et al.^24^ did not find any association between syndecan-1 immunoexpression and any of the clinicopathological data, including tumor grade, lymphovascular space invasion, lymph node metastasis, and tumor stage.

The current study did not show a significant association between syndecan-1 immunostaining and tumor grade, which contrasts with other studies.^11^,^12^,^25^ Few studies have evaluated the clinical outcomes in relation to syndecan-1 expression.^11^,^13^ In the current study, loss of syndecan-1 immunoexpression was an indicator of poor OS and DFS, which is similar to the results of one study,^11^,^12^ but contradicts those of another.^13^,^14^ Wang et al.^25^ evaluated syndecan-1 mRNA expression by RT-PCR in frozen CRC tissue and also found a decreased level to be associated with tumor size, tumor grade, depth of invasion, lymphovascular space invasion, lymph node metastasis, and TNM stage.

In a study by Wang et al., syndecan-1 mRNA expression was significantly higher in normal mucosa from the surgical margins of the specimen than in noncancerous mucosa adjacent to the CRC.^25^ Syndecan-1 shedding was found to be increased in CRC patients and decreased after chemotherapy.^26^ Patients with high serum level of syndecan-1 were found to be less responsive to chemotherapy.^26^ Syndecan-1 serum level was also shown to be a poor prognostic sign in CRC.^26^,^27^

Syndecans function as coreceptors or activators for molecules like growth factors and constituents of the matrix.^18^ Regarding syndecan-1’s regulation mechanism of cell attachment, it was suggested that it is a co-receptor of bFGF Type-1 growth factor, which has also shown decreased expression in CRC compared to adenoma, similarly to syndecan-1.^28^ Fujiya et al. suggest that a loss of syndecan-1 may weaken the signals that maintain the cellular differentiation of CRC cells and result in dedifferentiation and detachment from the extracellular matrix and adjacent cells.^12^

### Limitations of the study

The current study utilized only tissue microarray material, which includes representative cores of tissue. Normal mucosa adjacent to the tumor were not evaluated and this is considered limitation of the study.

## CONCLUSION

Loss of syndecan-1 immunoexpression is associated with metastatic potential, tumor recurrence and shorter survival in CRC and is considered a potential biomarker of poor prognosis in CRC patients. Nevertheless, further investigation of the role of syndecan-1 in CRC is required.

## References

[1] Bazarbashi S, Al Eid H, Minguet J (2017). Cancer Incidence in Saudi Arabia:2012 Data from the Saudi Cancer Registry. Asian Pac J Cancer Prev.

[2] Wei HT, Guo EN, Dong BG, Chen LS (2015). Prognostic and clinical significance of syndecan-1 in colorectal cancer:a meta-analysis. BMC Gastroenterol.

[3] Mitselou A, Galani V, Skoufi U, Arvanitis DL, Lampri E, Ioachim E (2016). Syndecan-1 Epithelial-Mesenchymal Transition Markers (E-cadherin/beta-catenin) and Neoangiogenesis-related Proteins (PCAM-1 and Endoglin) in Colorectal Cancer. Anticancer Res.

[4] Charchanti A, Papoudou Bai A, Samantas E, Papakostas P, Skarlos P, Kanavaros P (2019). Association of low Syndecan-1 expression with adverse histopathological parameters in gastric carcinomas. J Buon.

[5] Zeng Y, Yao X, Chen L, Yan Z, Liu J, Zhang Y (2016). Sphingosine-1-phosphate induced epithelial-mesenchymal transition of hepatocellular carcinoma via an MMP-7/ syndecan-1/TGF-beta autocrine loop. Oncotarget.

[6] Pasqualon T, Pruessmeyer J, Weidenfeld S, Babendreyer A, Groth E, Schumacher J (2015). A transmembrane C-terminal fragment of syndecan-1 is generated by the metalloproteinase ADAM17 and promotes lung epithelial tumor cell migration and lung metastasis formation. Cell Mol Life Sci.

[7] Wang X, He J, Zhao X, Qi T, Zhang T, Kong C (2018). Syndecan-1 suppresses epithelial-mesenchymal transition and migration in human oral cancer cells. Oncol Rep.

[9] Szarvas T, Sevcenco S, Modos O, Keresztes D, Nyirady P, Kubik A (2018). Circulating syndecan-1 is associated with chemotherapy-resistance in castration-resistant prostate cancer. Urol Oncol.

[10] Szarvas T, Reis H, Vom Dorp F, Tschirdewahn S, Niedworok C, Nyirady P (2016). Soluble syndecan-1 (SDC1) serum level as an independent pre-operative predictor of cancer-specific survival in prostate cancer. Prostate.

[11] Li K, Li L, Wu X, Yu J, Ma H, Zhang R (2019). Loss of SDC1 Expression Is Associated with Poor Prognosis of Colorectal Cancer Patients in Northern China. Dis Markers.

[12] Fujiya M, Watari J, Ashida T, Honda M, Tanabe H, Fujiki T (2001). Reduced expression of syndecan-1 affects metastatic potential and clinical outcome in patients with colorectal cancer. Jpn J Cancer Res.

[13] Hashimoto Y, Skacel M, Adams JC (2008). Association of loss of epithelial syndecan-1 with stage and local metastasis of colorectal adenocarcinomas:an immunohistochemical study of clinically annotated tumors. BMC Cancer.

[14] Mitselou A, Skoufi U, Tsimogiannis KE, Briasoulis E, Vougiouklakis T, Arvanitis D (2012). Association of syndecan-1 with angiogenesis-related markers, extracellular matrix components, and clinicopathological features in colorectal carcinoma. Anticancer Res.

[15] Amin MB, Edge S, Greene F, Byrd DR, Brookland RK, Gershenwald JE (2017). AJCC Cancer Staging Manual. https://www.springer.com/gp/book/9783319406176(Retrived19-9-2020).

[16] Al-Maghrabi J, Buhmeida A, Emam E, Syrjanen K, Sibiany A, Al-Qahtani M (2012). Cyclooxygenase-2 expression as a predictor of outcome in colorectal carcinoma. World J Gastroenterol.

[17] Al-Maghrabi J, Emam E, Gomaa W, Saggaf M, Buhmeida A, Al-Qahtani M (2015). c-MET immunostaining in colorectal carcinoma is associated with local disease recurrence. BMC Cancer.

[18] Lambaerts K, Wilcox-Adelman SA, Zimmermann P (2009). The signaling mechanisms of syndecan heparan sulfate proteoglycans. Curr Opin Cell Biol.

[19] Kim YI, Lee A, Lee BH, Kim SY (2011). Prognostic significance of syndecan-1 expression in cervical cancers. J Gynecol Oncol.

[20] Kim H, Choi DS, Chang SJ, Han JH, Min CK, Chang KH (2010). The expression of syndecan-1 is related to the risk of endometrial hyperplasia progressing to endometrial carcinoma. J Gynecol Oncol.

[21] Chen CL, Ou DL (2006). Expression of syndecan-1 (CD138) in nasopharyngeal carcinoma is correlated with advanced stage and poor prognosis. Hum Pathol.

[22] Mitselou A, Ioachim E, Peschos D, Charalabopoulos K, Michael M, Agnantis NJ (2007). E-cadherin adhesion molecule and syndecan-1 expression in various thyroid pathologies. Exp Oncol.

[23] Kim SY, Choi EJ, Yun JA, Jung ES, Oh ST, Kim JG (2015). Syndecan-1 expression is associated with tumor size and EGFR expression in colorectal carcinoma:a clinicopathological study of 230 cases. Int J Med Sci.

[24] Peretti T, Waisberg J, Mader AM, de Matos LL, da Costa RB, Conceicao GM (2008). Heparanase-2, syndecan-1, and extracellular matrix remodeling in colorectal carcinoma. Eur J Gastroenterol Hepatol.

[25] Wang H, Si JL, Zhang XZ, Qi YQ, Niu ZY, Zhou CH (2010). Expression and clinical significance of syndecan-1 mRNA and HPA-1 mRNA in colorectal cancer detected with real-time fluorescent quantitative polymerase chain reaction. Chin J Cancer.

[26] Wang X, Zuo D, Chen Y, Li W, Liu R, He Y (2014). Shed Syndecan-1 is involved in chemotherapy resistance via the EGFR pathway in colorectal cancer. Br J Cancer.

[27] Jary M, Lecomte T, Bouche O, Kim S, Dobi E, Queiroz L (2016). Prognostic value of baseline seric Syndecan-1 in initially unresectable metastatic colorectal cancer patients:a simple biological score. Int J Cancer.

[28] Volk R, Schwartz JJ, Li J, Rosenberg RD, Simons M (1999). The role of syndecan cytoplasmic domain in basic fibroblast growth factor-dependent signal transduction. J Biol Chem.

